# Understanding perceived determinants of nurses’ eating and physical activity behaviour: a theory-informed qualitative interview study

**DOI:** 10.1186/s40608-017-0154-4

**Published:** 2017-05-09

**Authors:** Brian T. Power, Kirsty Kiezebrink, Julia L. Allan, Marion K. Campbell

**Affiliations:** 1 School of Life and Medical Sciences, University of Hertfordshire, Hatfield, AL10 9AB UK; 2 Health Services Research Unit, University of Aberdeen, Health Sciences Building, Foresterhill, Aberdeen, AB25 2ZD UK; 3 Health Psychology, Division of Applied Health Sciences, University of Aberdeen, Aberdeen, AB25 2ZD UK

**Keywords:** Healthcare professionals, Diet, Exercise, Barriers, Enablers, Theoretical domains framework

## Abstract

**Background:**

Unhealthy eating and physical activity behaviours are common among nurses but little is known about determinants of eating and physical activity behaviour in this population. The present study used a theoretical framework which summarises the many possible determinants of different health behaviours (the Theoretical Domains Framework; TDF) to systematically explore the most salient determinants of unhealthy eating and physical activity behaviour in hospital-based nurses.

**Methods:**

Semi-structured qualitative interviews based on the TDF were conducted with nurses (*n* = 16) to explore factors that behavioural theories suggest may influence nurses’ eating and physical activity behaviour. Important determinants of the target behaviours were identified using both inductive coding (of categories emerging from the data) and deductive coding (of categories derived from the TDF) of the qualitative data.

**Results:**

Thirteen of the fourteen domains in the TDF were found to influence nurses’ eating and physical activity behaviour. Within these domains, important barriers to engaging in healthy eating and physical activity behaviour were shift work, fatigue, stress, beliefs about negative consequences, the behaviours of family and friends and lack of planning. Important factors reported to enable engagement with healthy eating and physical activity behaviours were beliefs about benefits, the use of self-monitoring strategies, support from work colleagues, confidence, shift work, awareness of useful guidelines and strategies, good mood, future holidays and receiving compliments.

**Conclusions:**

This study used a theory-informed approach by applying the TDF to identify the key perceived determinants of nurses’ eating and physical activity behaviour. The findings suggest that future efforts to change nurses’ eating and physical activity behaviours should consider targeting a broad range of environmental, interpersonal and intrapersonal level factors, consistent with a socio-ecological perspective.

**Electronic supplementary material:**

The online version of this article (doi:10.1186/s40608-017-0154-4) contains supplementary material, which is available to authorized users.

## Background

Many nurses display unhealthy eating and physical activity behaviours [1–4]. As an example, Tucker et al. (2010) report that half of nurses in Canada do not meet current recommendations for physical activity [5]. Further to this, approximately 50–65% of nurses are overweight or obese [6–9], a level consistent with prevalence rates in the general population. As obesity in nurses has been associated with a wide range of negative outcomes such as productivity loss, occupational injuries and musculoskeletal disorders [10–12] a weight management intervention specifically developed to change eating and physical activity behaviours in this group has considerable potential to be beneficial.

Recent systematic reviews have identified several weight management interventions targeting healthcare professionals [13, 14], however a major limitation of this field is that these interventions are not typically based on known theories of behaviour change. Theory-based interventions have been shown to be more likely to be effective [15] and critically, enable researchers to identify and build upon the ‘active ingredients’ of effective interventions [16].

To develop a theory-based intervention, the targets for intervention, that is the major determinants of the behaviours in question, must be identified. Determinants are factors that affect the nature or expression of a behaviour and can therefore hinder or enable behaviour change. They are frequently referred to as barriers and enablers [17]. In the present study, possible barriers and enablers of weight-related behaviours in nurses are identified through systematic application of the TDF. A small number of previous investigations have assessed the perceived determinants of nurses’ eating and physical activity behaviour but without using a comprehensive theoretical approach [18–21]. In one cross-sectional survey, lack of breaks, lack of canteen food selection, and canteen opening times were cited as barriers to nurses’ healthy eating [18]. Further, in a study of 394 Icelandic nurses, exhaustion and lack of time were reported as barriers to nurses’ physical activity [20].

Although these studies provide preliminary evidence about some of the possible determinants of nurses’ eating and physical activity behaviours, it is likely that many other determinants have gone unreported (e.g. those that were not spontaneously suggested as potentially relevant, that are less obvious or intuitive, or that the individual reporting believes could be perceived as a personal weakness or flaw). Further to this, although some evidence exists around the *barriers* to eating and physical activity behaviour in nurses, there is little evidence describing factors that enable nurses’ eating and physical activity behaviour. Eating and physical activity behaviours are likely to be a function of the complex interplay between individual, social and environmental influences [22]. One approach to capturing this complexity and to detect previously unreported barriers and enablers is to use a theoretical framework such as the ‘Theoretical Domains Framework’ (TDF), to systematically explore as many potential determinants of the behaviours in question as possible.

The present study uses the TDF to systematically explore the range of possible determinants of nurses’ eating and activity behaviour. The TDF is an overarching framework which synthesised 84 constructs from 33 different behavioural theories into 14 broad domains representing the core determinants of human behaviour [23] (illustrated in Table 1). Basing qualitative interviews on the TDF means that (a) a larger number of relevant determinants can be identified [24], (b) the relative importance of the different determinants can be compared, and (c) identified determinants can be mapped to appropriate behaviour change techniques (BCTs) through established mapping guidelines [25], thereby enabling the development of theory and evidence-based interventions.

**Table 1:** Theoretical domains framework (TDF) domains and associated definitions

Domain	Definition
Memory, attention and decision processes	The ability to retain information, focus selectively on aspects of the environment, and choose between two or more alternatives
Knowledge	An awareness of the existence of something
Skills	An ability or proficiency acquired through practice
Social Influences	Those interpersonal processes that can cause an individual to change their thoughts, feelings, or behaviours
Optimism	The confidence that things will happen for the best, or that desired goals will be attained
Social/professional role and identity	A coherent set of behaviours and displayed personal qualities of an individual in a social or work setting
Beliefs about capabilities	Acceptance of the truth, reality, or validity about an ability, talent, or facility that a person can put to constructive use
Beliefs about consequences	Acceptance of the truth, reality, or validity about outcomes of a behaviour in a given situation
Reinforcement	Increasing the probability of a response by arranging a dependent relationship, or contingency, between the response and a given stimulus
Intentions	A conscious decision to perform a behaviour or a resolve to act in a certain way
Goals	Mental representation of outcomes or end states that an individual wants to achieve
Environmental context and resources	Any circumstance of a person’s situation or environment that discourages or encourages the development of skills and abilities, independence, social competence, and adaptive behaviour
Behavioural regulation	Anything aimed at managing or changing objectively observed or measured actions
Emotion	A complex reaction pattern, involving experiential, behavioural, and physiological elements, by which the individual attempts to deal with a personally significant matter or event

## Methods

### Design

This was a semi-structured theoretically-informed qualitative interview study. Reporting of the study follows the relevant sections of the consolidated criteria for reporting qualitative research (COREQ) [26].

### Participants and recruitment

Any registered nurse with a permanent or temporary, part-time, or full-time position at a large teaching hospital in the North East of Scotland (Aberdeen Royal Infirmary) was eligible for inclusion. In addition to advertisements posted in hospital staffrooms, email invitations were circulated by each ward manager within the hospital to all registered nurses explaining the purpose of the study and providing the contact details of the researchers.

### Materials

A study-specific topic guide, based upon the TDF was developed for use in each qualitative interview, asking nurses about each of the 14 TDF domains shown in Table 1. The guide was study-specific as standard TDF interview questions were tailored to the topic of the current sudy. See Additional file 1 for the full interview topic guide.

### Procedure

Face-to-face semi-structured qualitative interviews were conducted between September, 2013 and November, 2013. All interviews were conducted by the first author (BTP) who at the time of the study was a doctoral researcher. The interviewer attended a one day qualitative research training course which provided an introduction to the theory and practice of qualitative interviewing. Further, the interviewer attended a three month training course in the Philosophy and Methods of Qualitative Sociology. This latter training course provided the interviewer with an opportunity to develop qualitative interviewing skills.

No relationship between the researcher and participants was established prior to study commencement. Participants were interviewed in a quiet location at the participants’ place of work with no other individuals present. Prior to participation, all participants provided informed, written consent. At the beginning of each interview, the participant was given the opportunity to choose between eating or physical activity behaviour for discussion. The choice made by the participant determined which of the two behaviours was explored in more detail for the remainder of the interview. This approach was taken to facilitate a more focussed data analysis. Elaboration and clarification probes [27] were used to explore issues in more depth. Data collection ceased when data saturation was assessed to have been reached [28]. Specifically, we adopted the “ten + three” stopping criterion advocated for use in theory-informed qualitative interviews [28], (i.e. a minimum of ten interviews plus subsequent interviews until three consecutive interviews with different participants produce no new ideas).

### Data analysis

Qualitative interviews were audio-recorded, transcribed verbatim and transferred into QSR-NVivo 10 for management. Following the procedures described by Mayring [29] and Elo and Kyngas [30], data were analysed using inductive coding (categories emerging from the data) and deductive coding (categories derived from the TDF) – explained below.

### Inductive category development

The first three interviews were inductively codedby two researchers independently (BTP, KK). This involvedbottom-up coding interview transcripts for the presence of beliefs about barriers and enablers to eating and physical activity. Beliefs coded were then organised into categories. This process resulted in the development of a data-driven coding manual consisting of categories, definitions of categories and rules for coding text with a category [31]. The coding manual developed was then applied to the analysis of each qualitative interview transcript and underwent refinements where appropriate to respond to emergent categories.

### Deductive category application

Following inductive category development in inductive content analysis, each main category was reviewed for content and allocated into relevant domains of the TDF (or recorded as not fitting into any of the TDF domains) by three researchers independently, BTP, KK, and JLA.

### Data summarisation

In addition to the inductive category development and deductive category application, the frequency with which each barrier/enabler was reported by participants was determined to enable identification of a TDF domains’ relative importance. This “quantitising” of qualitative data is widely used in healthcare research [32, 33]; and in the present study, enables the ‘modal salience’ of the domain to be identified [34, 35] i.e. the frequency with which specific beliefs within a domain were elicited, across the sample of transcripts as a whole, relative to other specific beliefs within other domains. Any frequently mentioned (i.e. beliefs that are mentioned by more than half of participants) were considered to be important for nurses’ eating and physical activity behaviour.

## Results

### Sample characteristics

**Fig. 1:**
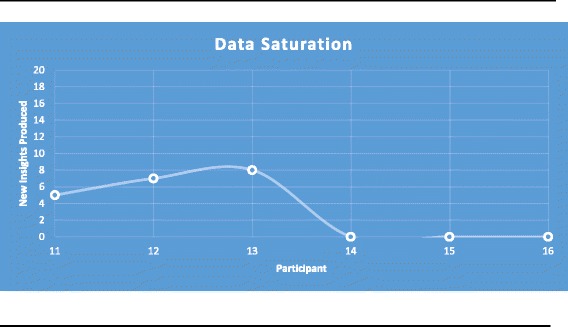
Data saturation using the 10 + 3 stopping criterion. Depicts themes being counted from interviewee 11 as for the purposes of data saturation only those past interview 10 are important (10 + 3 rule)

Data saturation was deemed to have been reached at interview sixteen (Fig. 1). The mean duration of interviews was 28 min (range 14–43 min). Participant characteristics are summarised in Table 2. The largest age category for participants was ≤30 years (*n* = 10). The second largest age subgroup was aged 31–40 years (*n* = 4), and there were two participants aged 41–50 years. Of the sixteen participants, six were a healthy weight, nine were overweight and one was obese. Three participants classified themselves as working “day shift only”, while thirteen described themselves working “day and night shift”.

**Table 2:** Characteristics of participants interviewed (*n* = 16)

Nurse	Age range	*BMI classification	Years nursing experience	Working shift pattern	Nursing band/grade
1	41-50	Healthy Weight	20+	Day shift only	6
2	31-40	Obese 1	6-10	Day and Night shift	5
3	≤30	Overweight	6-10	Day and Night shift	5
4	≤30	Overweight	6-10	Day shift only	6
5	31-40	Overweight	11-19	Day and Night shift	6
6	31-40	Overweight	11-19	Day and Night shift	6
7	≤30	Overweight	6-10	Day and Night shift	5
8	≤30	Healthy Weight	≤5	Day and Night shift	5
9	≤30	Overweight	6-10	Day and Night shift	5
10	≤30	Healthy Weight	6-10	Day and Night shift	5
11	≤30	Healthy Weight	6-10	Day and Night shift	6
12	31-40	Healthy Weight	6-10	Day and Night shift	5
13	≤30	Healthy Weight	≤5	Day and Night shift	5
14	≤30	Overweight	6-10	Day and Night shift	5
15	41-50	Overweight	20+	Day shift only	7
16	≤30	Overweight	≤5	Day and Night shift	5

*Calculated from self-reported weight and height

### Inductive category development

Categories generated through the inductive development process included motivation, strategies, fatigue, stress, past experiences, consequences, environment and confidence. The inductive content analysis did not generate any categories that could not be mapped to specific domains of the TDF. Specifically, the inductively developed categories were mapped to thirteen of the fourteen TDF domains. As such, the qualitative analysis was considered complete and the TDF was deemed to adequately capture all perceived determinants.

### Deductive category application

The perceived determinants of eating and physical activity behaviour among nurses that fall into each of the TDF domains and the frequency with which they were mentioned are illustrated in Table 3. Environmental context and resource factors (shift pattern, time, poor staffing levels, lack of breaks, cost, workplace policies and regulations and workplace food environment) were the most frequently quoted determinants of eating and physical activity behaviour by participants followed by beliefs about consequences. The least frequently quoted determinants of eating and physical activity behaviour included social, professional role and identity, optimism and skills.

**Table 3:** TDF domains identified as barriers/enablers to eating and physical activity

Domain sub categories generated from data	Utterance count			Nurses citing domain as barrier	Nurses citing domain as enabler
	Total utterances^a^	Barrier utterances^a^	Enabler utterances^a^		
Social influences	62	17	45	10/16	14/16
Family, friends, patients and work colleagues (±)	62	17	45		
Beliefs about capabilities	36	9	27	6/16	15/16
Confidence (±)	31	7	24		
Past experiences (±)	5	2	3		
Intentions	33	19	14	8/16	8/16
Lack of motivation (-)	19	19	0		
Having intentions (+)	14	0	14		
Knowledge	27	1	26	1/16	15/16
Awareness of guidelines and strategies (e.g. portion control, physical activity guidelines) (+)	25	0	25		
Awareness of local facilities (±)	2	1	1		
Goals	20	0	20	0/16	11/16
Priority (+)	6	0	6		
Holidays/Special occasions (+)	14	0	14		
Reinforcement	19	0	19	0/16	11/16
Immediate benefits (+)	16	0	16		
Compliments (+)	2	0	2		
Free gym membership (+)	1	0	1		
Social, Professional role and identity	16	2	14	4/16	8/16
Role model (+)	10	0	10		
Responsibilities of role as a nurse (±)	6	2	4		
Optimism	9	0	9	0/16	7/16
Positive about outcome of managing body weight (+)	9	0	9		
Skills	0	0	0	0/16	0/16
No subcategories from data					

(-) = barrier; (+) = enabler; (±) = conflicting beliefs whether barrier/enabler

^a^ = A belief whose content may indicate a perceived influence on eating and/or physical activity behaviour

## Environmental factors influencing nurses’ eating and physical activity behaviour

### Environmental context and resources

Nurses discussed the environment within which they managed their eating and physical activity, including specific contextual factors that could act as barriers to, or enablers of, their eating and physical activity behaviour. These factors related to policies and regulations in the workplace; role related tasks; the workplace food environment; workplace building layout; cost; poor staffing levels; lack of breaks; bad weather; food environment of workplace; availability of facilities; working shift pattern; equipment/transportation and time pressures.

It became evident during the interviews that the presence of unhealthy food in the workplace environment triggered overconsumption. The availability of “*sweeties*” given by patients’ relatives was an important issue making healthy eating behaviour difficult. Healthy food availability in the hospital was also described as “*limited*”. Additionally, the proximity to the limited healthy food within the hospital environment was reported as being “*quite a distance*”. As a consequence, nurses working night shift in particular were left to choose between “*chips or burgers or things like that*” [NURSE; 41–50 years; Overweight]. One nurse described resorting to eating “*a packet of crisps*” whilst working night shift due to this limited availability of healthy food in the workplace environment.

Nurses also indicated how financial considerations such as the *‘’expense of the canteen”* influenced whether they consumed healthy food. Limited economic resources forced some nurses to focus on the most pressing priorities, which in one particular case came at the expense of engaging in physical activity:

‘’*I’d much rather keep my you know my petrol money for getting to work rather than going somewhere else [the gym] that’s not you know essential*‘’ [NURSE; 31–40 years; Obese]

In relation to shift work, nurses conceptualised this with phrases such as “*long*”, “*busy*” and “*erratic*” and explained how it precipitated a “*hangover feeling*”. Nurses believed allocation of adequate breaks during working hours was an important prerequisite for successful eating behaviour as it would prevent “*pigging out*” at the end of a work shift. Despite this, nurses described being less likely to “*fit in breaks*”; more likely to “*go long spells without having anything*”; and more likely to “*grab a biscuit*” at home as a result of shift work. Conversely, some nurses mentioned certain shift patterns as potential enablers of their eating and physical activity behaviour. For example, working night shift enabled nurses to take advantage of going to the gym at less busy times of the day when other people were working.

## Interpersonal factors influencing nurses eating and physical activity behaviour

### Social influences

Many facets of social influences were discussed by nurses including family, friends, patients and work colleagues.

Some nurses considered family as key influencers of unhealthy eating and physical activity behaviours, and described how some family members made it difficult for them to make their eating and physical activity a priority. For instance, nurses living with a family expressed the need to include and balance the different preferences of family members which were sometimes at odds with instigating healthy eating behaviour patterns. Familial roles and responsibilities also emerged as a barrier to physical activity behaviour for some nurses. Nurses considered this sense of responsibility left them with less time to engage in physical activity.

Contrastingly, other nurses underscored the importance of their family as enablers of eating and physical activity behaviour. One nurse described how promoting healthy eating to their children acted as an enabling factor for their eating behaviour. Nurses also talked about supportive partners and how their involvement was helpful. The significance of having friends to turn to for “*support*” was also mentioned.

The family of patients were also cited as posing difficulty to nurses’ eating behaviour. One nurse captured this in the following comment:

‘’*Sweeties constantly being given by relatives*.‘’ [NURSE; 31–40 years; Overweight]

Nurse’s perceptions of their work colleagues’ support was an important trigger for certain eating and physical activity behaviours. Engaging in physical activity with a friend provided an opportunity to “*motivate each other*” and one nurse described how it “*spurs*” them on to participate in physical activity more frequently.

Although nurses reported receiving support from work colleagues, they found the influence of certain work colleagues problematic. For example, nurses commented on work colleagues bringing unhealthy food into work and how one particular colleague “*twists your arm until you take something”* [NURSE; ≤30 years; Overweight]. Nurses frequently ceded to this social pressure.

## Intrapersonal factors influencing nurses’ eating and physical activity behaviour

### Memory attention and decision processes

Concern related to “*distractions*” was explicitly shared by nurses in their interviews, and all described occasions in which their attention had been directed away from eating and physical activity behaviour. There was an expressed perception among most nurses that holidays’ and special occasions were distractions which led to disinhibited eating. Within the context of this study, disinhibition is defined as an uncontrolled overconsumption of food in response to a range of stimuli, such as cognitive/emotional states [36]. Nurses also mentioned distractions such as a desire to be ‘’*social*” and to “*savour*” their days off, as making eating and physical activity behaviour more difficult to achieve. Conversely, eating behaviour was considered easier to undertake if nurses were self-disciplined.

The inter-related nature of levels of influence is evidenced by the effects of environmental factors such as shift work on intrapersonal level factors such as memory, attention and decision processes. For instance; ten nurses attributed being “*exhausted*”, “*out of sorts*” or “*knackered*” as a result of shift work which subsequently affected their eating and physical activity behaviours. One nurse explained the relation between environmental influences and memory, attention and decision processes as follows;

“*Physical activity it just depends on energy wise…you know working your 12 h shifts it's pretty knackering. Em especially if you do 4, this is my 5th day so em I can't see myself doing much*” [NURSE; ≤30 years; Healthy Weight]

### Behavioural regulation

Several nurses had certain strategies and practices which they performed to manage their eating and physical activity behaviours. Specifically, nurses mentioned two behavioural strategies; self-monitoring and planning. Participants described using an “i*Phone so like I can measure how far I run and how quick I do it*” [NURSE; 31 – 40 years; Overweight], “*watching my weight*” and “*write things*” as useful forms of self-monitoring. One nurse revealed how self-monitoring provided visual representation of progress and areas for improvement:

“*I think you always kind of push yourself to do a bit better*” [NURSE; ≤30 years; Healthy Body Weight].

To “*fit*” eating and physical activity into their daily lives several nurses acknowledged the crucial enabling role that planning played.

### Beliefs about consequences

Many of the nurses reported that there were psychological and physical benefits of eating and physical activity behaviours that helped them. Nurses believed healthy eating behaviour made them “*less likely to go off sick or get stressed*”, gave them “*more energy*”, and helped them “*feel better*”. Nurses also reported beneficial outcomes of physical activity behaviour such as “*releasing a bit of frustration”,* “*alleviates stress”*, “*clear your mind”* and improving “*fitness level*” as key enablers of physical activity behaviour.

Negative consequences of physical activity such as being “*sweaty and gross*” were also viewed as important barriers by many nurses. Additionally, injury or concern about making an injury worse was cited as a barrier, as summarised by this nurse:

“*I've got a little bit of a bad back just from general wear and tear I think…and I.....actually just last a few weeks ago eh made appointment at the osteopath and he did some things so I didn't want to [do exercise], just in case there was damage…I didn't want to make it any worse* “[Nurse; ≤30 years; Overweight]

### Emotion

Nurses’ accounts revealed a range of difficulties that they experienced with emotion. These difficulties impacted on nurses’ eating and physical activity behaviour. Experiences of being “*stressed out*” inhibited thinking about healthy eating, as described in this account:

“*You immediately go for something sweet to try and make yourself feel better*” [NURSE; 31–40 years; Overweight]

Nurses attributed stress, in part, to lack of breaks and being busy;

“*You don’t get for your break maybe nine times out of ten because you’re so busy then you do get stressed out*” [NURSE; 31–40 years; Overweight]

Nurses also discussed occasions in which their mood hindered their eating behaviour and influenced them to “*eat junk*”. One nurse explained how experiences of low mood resulted in eating to “*comfort myself*”. Interestingly, nurses cited stress as both a barrier and enabler for physical activity behaviour. For example, a few nurses regarded stress related to the workplace as a barrier to physical activity. On the other hand, other nurses referred to stress experienced in the workplace as an enabling influence on physical activity behaviour. Nurses talked about how physical activity “*alleviates*” stress and provided an opportunity to *‘’clear your mind”.*

Nurses expressed conflicting views in relation to the influence of mood on their physical activity behaviour. Some nurses viewed mood as an inhibiting their physical activity behaviour. One nurse related their low mood to the death of patients’ under their care and described how it acted against her physical activity:

*‘’Sometimes you've had maybe a bad day in the sense that you've had maybe deaths and things and…you've been dealing with family and it's quite an emotional day so…You just want to go home and chill. You don't wanna go and well you don't think about oh I need to go for a run.‘’* [NURSE; ≤30 years; Overweight]

By contrast, the same nurse reported that on certain occasion’s mood enabled their physical activity as they considered physical activity helped them *“unwind”.*

### Beliefs about capabilities

Nurses reported confidence as a factor influencing their eating and physical activity behaviour. Concerns about their ability to change their eating behaviour due to their working shift pattern surfaced. For instance, eating healthily whilst on shift work was described as “*quite a struggle*”.

It became evident from nurses’ comments that their confidence or lack thereof could sometimes be traced back to past experiences. In relation to eating, some nurses recounted experiences of “*doing it in the past*” which resulted in them being “*pretty positive*” about their future ability to eat healthily. A remark by one nurse suggests failure in the past may act as an enabling factor for physical activity behaviour:

“*I've always had a problem with weight gain anyway so you know I have to do it for…for that or else the weight just goes on so easily so*” [NURSE; 31 – 40 years; Overweight]

### Goals

Most nurses reported eating and physical activity were important to them. Nurses recounted how eating and physical activity behaviour was “*high up there*” in terms of priorities. Reasons for eating and physical activity behaviour being important included wanting to “*lose weight*” for “*Christmas time*”, increasing chances “*for conceiving*”, to “*look nice for your other half*” and for certain “*special occasions”* a need to *“gear up”*.

### Reinforcement

Expectations of reward were seen to be an enabling reinforcement for eating and physical activity behaviour. As an example, one nurse noted how feeling *“chirpier”* enabled her to eat healthy. Analysis of the data also revealed how receiving compliments from others such as *“you look like you’ve lost weight”* was an important enabler of physical activity behaviour.

### Knowledge

Many nurses discussed their knowledge as helpful with eating and physical activity behaviour. Despite having the facts, information, and awareness of what eating and physical activity behaviour requires, other remarks suggest this is a necessary but insufficient factor for changing behaviour:

“*I know what I need to do. It's just doing that*” [NURSE; 31–40 years; Overweight]

### Optimism

Beliefs that goals will be achieved was verbalised by seven nurses as a key enabler of their eating and physical activity behaviours. One nurse expressed this optimism as follows;

*“I think it will be very successful because like you know when I exercise I see effects quite quickly”* [NURSE; ≤30 years; Healthy weight]

## Discussion

The current study goes beyond previously published investigations in three ways. Firstly, through the elicitation of qualitative information about barriers and enablers of eating and physical activity behaviour among nurses; secondly by assessing the relative importance of these barriers and enablers and thirdly, in applying a theoretical approach to structure data collection and analysis and inform future intervention development recommendations. Six TDF domains were perceived as particularly important barriers to eating and physical activity behaviour: “Environmental context and resources”, “memory, attention and decision processes”, “emotion”, “beliefs about consequences”, “behavioural regulation” and “social influences”. Nine TDF domains were perceived as particularly important enablers of nurses’ eating and physical activity behaviour and included the TDF domains; “beliefs about consequences”, “behavioural regulation”, “environmental context and resources”, “social influences”, “beliefs about capabilities”, “knowledge”, “emotion”, “goals” and “reinforcement”.

Taking the above into account, the current study indicates that barriers to and enablers of nurses’ eating and physical activity behaviour operate at different levels – environmental, interpersonal and intrapersonal. Using the TDF provided a window into the interplay of these environmental, interpersonal and intrapersonal determinants simultaneously within one study. For example, the current study illustrated the apparent relationship between determinants within the environmental context and resources domain. Specifically, staff shortages exacerbated time constraints experienced by nurses, contributed to an increased demand on nurses' schedules and impacted on their capacity to take breaks. Additionally, although it has been previously identified that environmental factors make eating behaviour difficult [37], this study provided insight into exactly how these environmental factors may interact with other factors such as memory, attention and decision processes to influence eating and physical activity behaviour. For example, ten nurses attributed being exhausted or out of sorts to shift work which subsequently affected their eating and physical activity behaviours. The interrelated nature of determinants across different levels suggests that interventions which address one level may produce knock on effects on perceived determinants operating at a different level.

Another benefit of classifying barriers and enablers into TDF domains lies in the opportunity it provides to identify appropriate behaviour change techniques (BCTs) for targeting particular determinants. BCTs are observable, replicable, and irreducible active ingredients of behaviour change interventions that on their own have the potential to bring about change in behaviour (e.g. self-monitoring and goal setting). Twelve of the TDF domains have already been mapped to 59 BCTs from a BCT Taxonomy according to their ‘usefulness’ for influencing determinants in each TDF domain [25]. The BCT taxonomy is a 93-item taxonomy of BCTs designed to improve the reporting and scientific study of behaviour change interventions. This mapping could be used in future to identify the most promising BCTs for inclusion in a weight management intervention for nurses as the present study identifies the TDF domains which should be targeted for change.

Findings from the current study also contribute to the evidence base by explaining why some nurses are more successful than others in managing their eating and physical activity behaviours despite sharing the same working environment. The study revealed nurses’ perceptions about barriers and enablers to eating and physical activity behaviour can be extremely diverse. For example, what constitutes a barrier or enabler differed from nurse to nurse. While on the one hand environmental context and resources was the most frequently mentioned perceived barrier by nurses, on the other, some nurses perceived certain environmental context and resources factors as facilitating healthy eating and physical activity behaviour. For example, certain nurses suggested working night shift offered them the opportunity to participate in physical activity during daytime hours. In line with this finding, previous research has found that many nurses consider working night shift favourable as it offers flexibility and freedom during the day not afforded to nurses’ working day shift [38, 39]. In contrast, other interviewed nurses perceived their working shift pattern as a barrier to engaging in physical activity as it encroached upon spare time. This mirrors previously discussed findings within studies of office workers [40]. Additionally, a systematic review by Amani and Gill (2013) examined eating behaviour among shift workers in factory-based settings and demonstrated that shift work acted as a barrier to healthy eating behaviour of these employees [41]. The reasons for these differing beliefs in the current study may be related to different life stages, e.g. certain nurses having children which may place additional demands on their time.

Emotional influences such as stress, in particular work-related stress, were also described as having differential effects on eating and physical activity behaviour of nurses. Specifically, for most nurses, work related stress was perceived as a barrier to healthy eating; whereas stress was perceived as both detrimental and helpful towards participation in physical activity. In relation to eating, stress driven eating is widely considered as contributing to weight gain [42, 43]. Stress induced eating among nurses may be psychologically driven, whereby eating offers comfort and relief from a preceding aversive experience [44]. Previous research has also revealed that individuals who use food as comfort frequently report difficult childhood family relationships characterised by loneliness [45]. Using food as comfort in response to stress may therefore be associated with pleasurable situations and reward (as opposed to nutritional need) via early learning experiences [46]. As such, comfort eating may be a learned coping mechanism to manage experiences of emotional pain and stress, by providing a sense of security. Our finding that stress is associated with unhealthy eating practices, resonates with several past studies in occupational groups such as public administration employees [47] and nurses [48–50]. For instance, in the UK, the Nurses’ Health Study revealed that those who reported higher stress levels snacked on biscuits, chocolate, and crisps more frequently than those who were less stressed [51]. Other studies, in contrast, have produced different results, with some reporting that food intake remains unchanged [52], or decreases [53] in response to stress situations.

Regarding physical activity and stress, findings indicate that a multifarious relationship exists between stress and nurses’ physical activity. There are many possible explanations for individual differences in physical activity in response to stress among nurses. It may be a function of the general coping style of the nurses interviewed. As an example, under conditions of stress, those with a rigid coping style may be more inclined to increase physical activity behaviour [54].

The TDF domain ‘’memory, attention and decision processes” was mentioned frequently as having an influence on eating and physical activity behaviour. In particular, fatigue was perceived as being a hindrance to healthy eating and physical activity behaviour. Fatigue can affect quality of life through reductions in work performance and activities with peers, emotional functioning and general health [55]. Fatigue can also affect well-being and functional capacity resulting in reduced participation in physical activity and lack of motivation to follow healthy eating [56]. Further, it has been revealed that nurses who work shift patterns, including night shift, are prone to decreased quantity and quality of sleep, cumulative sleep debt, and constant sleep deprivation, thereby aggravating fatigue [57]. These findings may in part, explain how low sleep duration related to working night shift contributes to an elevated risk of overweight and obesity [58].

Nurses perceived friends and work colleagues as playing important enabling roles in their eating and physical activity behaviours. Social support has been previously reported to help office-based workers improve their physical activity behaviour [59]. Social desirability, social approval and informational social influence processes are possible mechanisms that may explain how eating norms influence nurses’ eating behaviour [60–62]. Social interaction, companionship, group cohesion and positive reinforcement are potential mechanisms through which social influences shape physical activity [63, 64].

In addition to friends and peers, many nurses also perceived family members, particularly partners, as positively influencing eating and physical activity behaviours. This corroborates evidence from previous studies that highlight how eating and physical activity behaviour changes made by one partner are often incorporated into the eating and physical activity choices of the other partner [65]. As an example, it has been shown that females respond to male partner influence by changing the quality of their food intake [66] and levels of physical activity [67]. Other work also suggests partners become important social influences for health in adulthood [68]. For instance, during the transition to motherhood, there is evidence indicating husband support is a particularly important influence on women remaining physically active [68]. Taken together, these findings further reinforce the idea that social support networks are crucial in shaping health behaviours [69] and that weight loss clusters in social networks [70].

### Strengths and limitations

While most research in this area has examined barriers [18, 19] and [71], fewer studies have investigated both barriers and enablers, limiting insight into enablers of healthy eating and physical activity behaviour in nurses. Also, no previous study has reported an exploration of both barriers and enablers to nurses’ eating and physical activity using a theoretical approach to structure data collection and analysis. There were no findings inductively analysed that could not be coded to one or more domains of the TDF, supporting the relevance of the TDF to health behaviours such as eating and physical activity among nurses. Both inductive (‘bottom up’) and deductive (‘top down’) content analyses were used in this study to analyse the interview data rather than use of a single approach. This allowed important categories to emerge that were not guided by the TDF. Furthermore, it enabled us to corroborate categories and therefore confirm our findings [72]. Multiple coding of interview transcripts improved the quality of content analysis and increased the dependability of interpretations. Dependability relates to the stability of the data that emerged over time or conditions [73, 74].

This study has some limitations however. Firstly, data presented in this study represent the *perceptions* of nurses interviewed and, as such, may not represent actual influences on nurses’ eating and physical activity behaviour in practice. Indeed, a significant body of emerging literature suggests that people make approximately 200 eating-related decisions per day with little or no conscious awareness [75]. Put another way, individuals may not have insight into many of the factors which influence their behaviour. Actual influences that may guide eating behaviour include hunger, food habits and exposure to food promotion [76]. Conducting a prospective observational study of nurses using an ecological momentary assessment method [77] may be one prudent option to overcome this limitation. Such a method would identify determinants more objectively than self-reporting qualitative interviews and survey methods. Social desirability bias may also have influenced nurses’ accounts of determinants of their eating and physical activity.

## Conclusions

The current study is an essential first step towards identifying determinants affecting nurses’ eating and physical activity behaviour and adds to the limited body of evidence in this area. Determinants which are particularly important for nurses but which have been under-acknowledged in previous investigations have been identified. These include a number of determinants that operate at the intrapersonal level such as behavioural regulation, beliefs about consequences, knowledge and optimism. Findings from this study suggest nurses’ eating and physical activity behaviour may be more effectively sustained if multiple levels of influence are targeted concurrently. Having identified the most important determinants of nurses’ eating and physical activity behaviour, a basis for considering how to change these target behaviours has been established. Accordingly, the next step for this work will involve identifying what behaviour change techniques could be used to change eating and physical activity behaviour among nurses.

## Additional file

Additional file 1: Interview topic guide. (DOCX 46 kb)

## Abbreviations

BCT: Behaviour change technique
COREQ: Consolidated criteria for reporting qualitative research
TDF: Theoretical domains framework

## References

[1] Cheung ST (2003). The effects of chocolates given by patients on the well-being of nurses and their support staff. Nutr Health.

[2] Jinks AM, Lawson V, Daniels R (2003). A survey of the health needs of hospital staff: Implications for health care managers. J Nurs Manag.

[3] Blake H, Mo PKH, Lee S, Batt ME (2012). Health in the NHS: Lifestyle behaviours of hospital employees. Perspect Public Health.

[4] Blake H, Patterson J (2015). Paediatric nurses' attitudes towards the promotion of healthy eating. Br J Nurs.

[5] Tucker SJ, Harris MR, Pipe TB, Stevens SR (2010). Nurses' ratings of their health and professional work environments. AAOHN J.

[6] Zapka J, Lemon SC, Estabrook BB, Jolicoeur DG (2007). Keeping a step ahead: Formative phase of a workplace intervention trial to prevent obesity. Obesity.

[7] Han K, Trinkoff AM, Storr CL, Geiger-Brown J (2011). Job stress and work schedules in relation to nurse obesity. J Nurs Adm.

[8] Bogossian FE, Hepworth J, Leong GM, Flaws DF, Gibbons KS, Benefer CA (2012). A cross-sectional analysis of patterns of obesity in a cohort of working nurses and midwives in Australia, New Zealand, and the United Kingdom. Int J Nurs Stud.

[9] Kyle RG, Neall RA, Atherton IM (2015). Prevalence of overweight and obesity among nurses in Scotland: A cross-sectional study using the Scottish Health Survey. Int J Nurs Stud.

[10] Letvak S, Ruhm C, Gupta S (2013). Differences in health, productivity and quality of care in younger and older nurses. J Nurs Manag.

[11] Jordan G, Behnam NK, Basem G, Behdin N (2015). Obesity as a Possible Risk Factor For Lost-Time Injury In Registered Nurses: A Literature Review. Saf Health Work.

[12] Reed LF, Battistutta D, Young J, Newman B (2014). Prevalence and risk factors for foot and ankle musculoskeletal disorders experienced by nurses. BMC Musculoskelet Disord.

[13] Chan CW, Perry L (2012). Lifestyle health promotion interventions for the nursing workforce: A systematic review. J Clin Nurs.

[14] Power BT, Kiezebrink K, Allan JL, Campbell MK (2014). Effects of workplace-based dietary and/or physical activity interventions for weight management targeting healthcare professionals: a systematic review of randomised controlled trials. BMC Obesity.

[15] Taylor N, Conner M, Lawton R (2012). The impact of theory on the effectiveness of worksite physical activity interventions: A meta-analysis and meta-regression. Health Psychol Rev.

[16] Prestwich A, Webb TL, Conner M (2015). Using theory to develop and test interventions to promote changes in health behaviour: Evidence, issues, and recommendations. Curr Opin Psychol.

[17] Krause J, Van Lieshout J, Klomp R, Huntink E, Aakhus E, Flottorp S (2014). Identifying determinants of care for tailoring implementation in chronic diseases: An evaluation of different methods. Implement Sci.

[18] Faugier J, Lancaster J, Pickles D, Dobson K. Barriers to healthy eating in the nursing profession: Part 1. Nurs Stand. 2001a;15:33-36. 10.7748/ns2001.05.15.36.33.c3030 12205837

[19] Faugier J, Lancaster J, Pickles D, Dobson K. Barriers to healthy eating in the nursing profession: Part 2. Nurs Stand. 2001b;15:33-35. 10.7748/ns2001.05.15.37.33.c3033 12205763

[20] Sveinsdottir H, Gunnardsdottir HK (2008). Predictors of self-assessed physical and mental health of Icelandic nurses: Results from a national survey. Int J Nurs Stud.

[21] Albert NM, Butler R, Sorrell J (2014). Factors related to healthy diet and physical activity in hospital-based clinical nurses. Online J Issues Nurs.

[22] Cohen DA, Babey SH (2012). Contextual influences on eating behaviours: Heuristic processing and dietary choices. Obes Rev.

[23] Cane J, O’Connor D, Michie S (2012). Validation of the theoretical domains framework for use in behaviour change and implementation research. Implement Sci.

[24] Francis JJ, O’Connor D, Curran J (2012). Theories of behaviour change synthesised into a set of theoretical groupings: introducing a thematic series on the theoretical domains framework. Implement Sci.

[25] Cane J, Richardson M, Cane J, Johnston M, Ladha R, Michie S (2015). From lists of behaviour change techniques (BCTs) to structured hierarchies: Comparison of two methods of developing a hierarchy of BCTs. Br J Health Psychol.

[26] Tong A, Sainsbury P, Craig J (2007). Consolidated criteria for reporting qualitative research (COREQ): a 32-item checklist for interviews and focus groups. Int J Qual Health Care.

[27] Patton M (2002). Qualitative evaluation and research methods.

[28] Francis JJ, Johnston M, Robertson C, Glidwell L, Entwistle V, Eccles MP (2010). What is an adequate sample size? Operationalising data saturation for theory-based interview studies. Psychol Health.

[29] Mayring P. Qualitative Content Analysis. Forum: Qualitative Social Research. 2000;1(2). http://nbn-resolving.de/urn:nbn:de:0114-fqs0002204. Accessed 11 Jan 2016.

[30] Elo S, Kyngas H (2008). The qualitative content analysis process. J Adv Nurs.

[31] Weber RP (1990). Basic Content Analysis.

[32] Sandelowski M (2000). Focus on research methods: Whatever happened to qualitative description?. Res Nurs Health.

[33] O’Cathain A, Murphy E, Nicholl J (2010). Three techniques for integrating data in mixed methods studies. BMJ (Online).

[34] Francis JJ, Stockton C, Eccles MP, Johnston M, Cuthbertson BH, Grimshaw JM, Hyde C, Tinmouth A, Stanworth SJ (2009). Evidence-based selection of theories for designing behaviour change interventions: Using methods based on theoretical construct domains to understand clinicians' blood transfusion behaviour. Br J Health Psychol.

[35] Islam R, Tinmouth AT, Francis JJ, Brehaut JC, Born J, Stockton C (2012). A cross-country comparison of intensive care physicians' beliefs about their transfusion behaviour: A qualitative study using the theoretical domains framework. Implement Sci.

[36] Carraca EV, Silva MN, Markland D, Vieira PN, Minderico CS, Sardinha LB (2012). Body image change and improved eating self-regulation in a weight management intervention in women. Int J Behav Nutr Phys Activ.

[37] McNaughton SA, Crawford D, Ball K, Salmon J (2012). Understanding determinants of nutrition, physical activity and quality of life among older adults: the Wellbeing, Eating and Exercise for a Long Life (WELL) study. Health Qual Life Outcomes.

[38] West S, Boughton M, Byrnes M (2009). Juggling multiple temporalities: The shift work story of mid-life nurses. J Nurs Manag.

[39] Hagger MS, Wood C, Stiff C, Chatisarantis NLD (2010). Ego Depletion and the Strength Model of Self-Control: A Meta-Analysis. Psychol Bull.

[40] Edmunds S, Hurst L, Harvey K (2013). Physical activity barriers in the workplace. Int J Workplace Health Manage.

[41] Amani R, Gill T (2013). Shiftworking, nutrition and obesity: implications for workforce health- a systematic review. Asia Pac J Clin Nutr.

[42] Allom V, Mullan B (2014). Individual differences in executive function predict distinct eating behaviours. Appetite.

[43] Berset M, Semmer NK, Elfering A, Jacobshagen N, Meier LL (2011). Does stress at work make you gain weight? a two-year longitudinal study. Scand J Work Environ Health.

[44] Finch LE, Tomiyama AJ (2015). Comfort eating, psychological stress, and depressive symptoms in young adult women. Appetite.

[45] Stroebe W, Papies EK, Aarts H (2008). From homeostatic to hedonic theories of eating: Selfregulatory failure in food-rich environments. Appl Psychol An Int Rev.

[46] Potocka A, Moscicka A (2011). Occupational stress, coping styles and eating habits among Polish employees. Med Prev.

[47] Sproesser G, Schupp HT, Renner B (2014). The Bright Side of Stress-Induced Eating: Eating More When Stressed but Less When Pleased. Psychol Sci.

[48] Ng DM, Jeffery RW (2003). Relationships between Perceived Stress and Health Behaviors in a Sample of Working Adults. Health Psychol.

[49] KivimakiI M, Head J, Ferrie JE, Shipley MJ, Brunner E, Vahtera J (2006). Work stress, weight gain and weight loss: Evidence for bidirectional effects of job strain on body mass index in the Whitehall II study. Int J Obes.

[50] Chaplin K, Smith AP (2011). Breakfast and snacks: Associations with cognitive failures, minor injuries, accidents and stress. Nutrients.

[51] Stroud LR, Tanofsky-Kraff M, Wilfley DE, Salovey P (2000). The Yale Interpersonal Stressor (YIPS): Affective, physiological, and behavioral responses to a novel interpersonal rejection paradigm. Ann Behav Med.

[52] Torres SJ, Nowson CA (2007). Relationship between stress, eating behavior, and obesity. Nutrition.

[53] Twisk JWR, Snel J, Kemper HCG, Van Mechelen W (1999). Changes in daily hassles and life events and the relationship with coronary heart disease risk factors: a 2-year longitudinal study in 27–29- year-old males and females. J Psychosom Res.

[54] Seigel K, Broman J, Hetta J (2002). Behavioral activation or inhibition during emotional stress - Implications for exercise habits and emotional problems among young females. Nord J Psychiatry.

[55] McVicar A (2003). Workplace stress in nursing: A literature review. J Adv Nurs.

[56] Muecke S (2005). Effects of rotating night shifts: Literature review. J Adv Nurs.

[57] Lyytikainen P, Rahkonen O, Lahelma E, Lallukka T (2011). Association of sleep duration with weight and weight gain: a prospective follow-up study. J Sleep Res.

[58] Herman CP, Roth DA, Polivy J (2003). Effects of the Presence of Others on Food Intake: A Normative Interpretation. Psychol Bull.

[59] Mazzola JJ, Taylor MJ, Alexander K. Is work keeping us from acting healthy? How workplace barriers and facilitators impact nutrition and exercise behaviors. Stress Health. 2016. doi: 10.1002/smi.2731 10.1002/smi.2731 27891758

[60] Ball K, Jeffery RW, Abbott G, McNaughton SA, Crawford D (2010). Is healthy behavior contagious: Associations of social norms with physical activity and healthy eating. Int J Behav Nutr Phys Act.

[61] Robinson E, Thomas J, Aveyard P, Higgs S (2014). What everyone else is eating: a systematic review and meta-analysis of the effect of informational eating norms on eating behavior. J Acad Nutr Diet.

[62] Hogan BE, Linden W, Najarian B (2002). Social support interventions: do they work?. Clin Psychol Rev.

[63] Shelton RC, McNeill LH, Puleo E, Wolin KY, Emmons KM, Bennett GG (2011). The association between social factors and physical activity among low-income adults living in public housing. Am J Public Health.

[64] Homish GG, Leonard KE (2008). Spousal influence on general health behaviors in a community sample. Am J Health Behav.

[65] Skoyen JA, Blank E, Corkery SA, Butler EA (2013). The interplay of partner influence and individual values predicts daily fluctuations in eating and physical activity. J Soc Pers Relationships.

[66] Jackson SE, Steptoe A, Wardle J (2015). The influence of partner's behavior on health behavior change: The English longitudinal study of ageing. JAMA Intern Med.

[67] Umberson D, Crosnoe R, Reczek C (2010). Social relationships and health behavior across the life course. Annu Rev Sociol.

[68] McIntyre CA, Rhodes RE (2009). Correlates of leisure-time physical activity during transitions to motherhood. Women Health.

[69] Laranjo L, Arguel A, Neves AL (2014). The influence of social networking sites on health behavior change: a systematic review and meta-analysis. J Am Med Inform Assoc.

[70] Leahey TM, Larose JG, Fava JL, Wing RR (2011). Social influences are associated with BMI and weight loss intentions in young adults. Obesity.

[71] Phiri LP, Draper CE, Lambert EV, Kolbe-Alexander TL (2014). Nurses’ lifestyle behaviours, health priorities and barriers to living a healthy lifestyle: a qualitative descriptive study. BMC Nurs.

[72] Peters S (2010). Qualitative research methods in mental health. Evid Based Ment Health.

[73] Crabtree BF, Miller WL (1999). Doing qualitative research.

[74] Sweeney A, Grenwood KE, Williams S, Wykes T, Rose DS (2013). Hearing the voices of service user researchers in collaborative qualitative data analysis: The case for multiple coding. Health Expect.

[75] Wansink B, Sobal J (2007). Mindless eating: The 200 daily food decisions we overlook. Environ Behav.

[76] Symmank C, Mai R, Hoffmann S, Stok FM, Renner B, Lien N, Rohm H (2017). Predictors of food decision making: A systematic interdisciplinary mapping (SIM) review. Appetite.

[77] Smyth J, Wonderlich S, Crosby R, Miltenberger R, Mitchell J, Rorty M (2001). The use of ecological momentary assessment approaches in eating disorder research. Int J Eat Disord.

