# Supplementary material for: Understanding perceived determinants of nurses’ eating and physical activity behaviour: a theory-informed qualitative interview study
Source: BMC Obes. 2017 May 9;4:18. doi: 10.1186/s40608-017-0154-4 (PMC5422972; doi:10.1186/s40608-017-0154-4)
Supplement: Additional file 1: — Interview topic guide. (DOCX 46 kb) [file 40608_2017_154_MOESM1_ESM.docx]

# Additional File 1. Qualitative interview topic guide

**** **
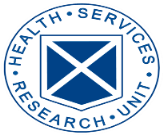
**

**INTERVIEWER INSTRUCTIONS**

The theoretical domains (and associated constructs) that underpin the questions and guide the focus of the interview are stated and briefly described. Each interview will be conducted in a conversational style, and the exact wording and order of questions about these topics will vary depending on what the respondents say and how. Interviewer will use careful ‘probe’ questions to ask (for example) for clarification, further details, illustrative examples to help gain an in-depth understanding of participants’ views.

The behaviours to be explored are specified according to the TACT principle—in terms of its Target, the Action to be performed, the Context or conditions for the action and when the action would be performed (Time). In this study, for nurses’ eating and physical activity behaviour change, the target is nurses, the action is eating and physical activity, the context is in the workplace and out of the workplace, and the time is during work time and leisure time.

**START OF INTERVIEW**

I would first like to thank you for taking the time to speak with me today. I am Brian and I am a PhD student studying how people in general and healthcare professionals like you in particular go about managing their eating and physical activity behaviours. By managing eating and physical activity behaviours I mean keeping these behaviours at a healthy level. The aim of this interview is to get a better understanding of how working as a healthcare professional may influence your eating and physical activity choices. I want to find out what your views, opinions and experiences are on this. The discussion is about what you believe and your experiences - there are no right or wrong answers. How does that sound? Are you ready to get started?

**INTRODUCING QUESTIONS**

**I’d like to start with some basic questions about you:**

Male or Female (to keep track of, won’t be asked)

1. How long have you been a nurse? (years) 1a What grade nurse are you?

2. How long have you been nurse in this particular hospital? (years)

3. What type of nursing speciality do you work in? (Environmental context)

4. Do you work day or night shift? (Environmental context)

Thanks very much for this.

5. Would you like to discuss eating or physical activity behaviour change in more detail for the rest of the interview?

Bearing this in mind, for the rest of the interview I have some slightly more specific questions to try and explore potential influences on _____________________________ (insert the behaviour the nurse selects).

| **Domain** | **Component constructs** | **Core Question** | **Possible Prompts** |
| --- | --- | --- | --- |
| **Environmental context and resources** | *Resources / material resources* | 6. In what way do you think your job affects your ________? | Would you explain that further? |
|  | *Environmental stressors* | 7. To what extent do aspects of your environment hinder your _________? Help? | Physical vs. resource factors  Clinical/home environment |
|  | *Barriers and facilitators*  *Organisational culture /climate*  *Salient events / critical incidents*  *Person x environment interaction* | 8. Can you think of other aspects of your job that hinder your ________? Or help? | Policies; attitudes/culture  Can you tell me of an example of when this happened? |
| **Social/Professional role & identity** | \| Professional role \| \| --- \| | 9. Would you view _________ as compatible or in conflict with your role as a nurse? | Could you provide me with more details on that? |
| **Social influences** | *Social pressure*  *Social support*  *Social norms* | 10. In your view, do other team members influence your _____________? Hinder or help?  11. How do the views/opinions/expectations of patients, relatives, and the general public affect your __________? Hinder or help? | Who else? Nursing colleagues, managers, other healthcare professionals.  In what circumstances? To what extent? |
| **Beliefs about capabilities** | *Self confidence* | 12. How confident are you about ____________? | Would you explain that further? |
|  | *Self-efficacy*  *Perceived behavioural control* | 13. What, if any, problems have you encountered in _________________? | Internal and external capabilities/constraints  Can you give me an example? |
| **Domain** | **Component constructs** | **Core Question** | **Possible Prompts** |
| **Beliefs about consequences** | *Beliefs*  *Outcome expectancies*  *Anticipated regret* | 14. How about advantages associated with____________ for you – what are your views on this?  Do you think there might be some, and if so what might these be? How about disadvantages? | To patients, colleagues and the organisation also; positive and negative, short term and long term consequences |
| **Goals** | *Goal priority*  *Goals (autonomous / controlled) Goal / target setting Action planning* | 15. How much do you feel you want to _________? | Can you elaborate on that? |
|  | *Goal priority* | 16. How important is _________ for you?  17. How does _____________ fit with other priorities you have? | What do you think are some reasons for that?  Priorities and goals that are incompatible with __________; priorities and goals that are in line with ___________? |
| **Memory, Attention and Decision Processes** | *Decision making* | 18. Would you say __________- is something you do automatically or something you would have to take time to think about doing? | What do you think are some reasons for that? |
|  | *Memory*  *Decision making*  *Decision making Cognitive overload / tiredness* | 19. What triggers for remembering ___________do you have? | What goes through your mind thinking about xyz factors?  Time of day |
|  | *Attention*  *Attention control* | 20. In what situations, if any, would you forget __________? | What do you think are some reasons for that? |
| **Domain** | **Component constructs** | **Core Question** | **Possible Prompts** |
| **Emotion** | *Affect* | 21. How does the way you feel affect your ________________?  22. Would you think this hinders your ______________? Or helps? | Can you describe situations in which this happened? |
|  | *Stress* | 23. How would you describe the influence of stress on your __________-?  24. Would you think this hinders your ____________?  Or helps? | If possible, please describe examples. |
| **Behavioural regulation** | *Breaking habit Self-monitoring Action planning* | 25. If you wanted to change your own ___________-, how would you do this? | What would need to happen? |
|  | *Breaking habit Self-monitoring Action planning* | 26. Within your workplace are there procedures or ways of working that encourage ____________?  Anything make it challenging? |  |
| **Intentions** | *Stability of intentions* | 27. Do you intend (to continue) __________? | If yes, Do you anticipate any problems? If no, What do you think are some reasons for that? |
| **Skills** | *Skills* | 28. How easy or difficult do you find ___________ ?  29. When working as a nurse, what specific skills, if any, do you think are needed for _____? | What makes it easy or difficult for you? |
| **Optimism** | *Optimism*  *Pessimism* | 30. In your opinion, how likely or unlikely is it that your__________ will be successful? | Would you explain that further? |
| **Reinforcement** | *Rewards (proximal / distal, valued / not valued, probable / improbable) Incentives* | 31. Would you say there is anything that would make you more or less likely to_____? | Incentives. What do you think are some reasons for that? |
| **Other** | *n/a* | 32. Is there anything else important that we have not already talked about that you think would help me understand this topic better? |  |
| **Secondary focus**  Participation in a workplace dietary and physical activity intervention | *n/a* | 33. The purpose of this study is not to recruit you to a trial but if there was a study which randomised nurses to a nutrition and physical activity intervention against a no-intervention control group would you be willing to participate? |  |

Can I ask you a few last things before ending the interview?

**CHECKLIST FOR DIVERSITY SAMPLING**

|  | **Date** |  |
| --- | --- | --- |
|  | **Participant ID** |  |
|  | **Question** | **Response** |
| 34. | Age range <30, 31-40, 41-50, 51-60, 61+ |  |
| 35. | (Self-reported Height/Weight to calculate BMI) |  |

**Conclusion**

Thanks for your time and valuable input, that is me finished with my questioning.

**CLOSE OF INTERVIEW**
